# Health-related quality of life, workability, and return to work of patients after liver transplantation

**DOI:** 10.1007/s00423-021-02183-z

**Published:** 2021-05-06

**Authors:** Christian Fazekas, Daniela Kniepeiss, Nora Arold, Franziska Matzer, Jolana Wagner-Skacel, Peter Schemmer

**Affiliations:** 1 Department of Medical Psychology and Psychotherapy, Medical University of Graz, Graz, Austria; 2 Transplant Center Graz, Graz, Austria; 3 General, Visceral and Transplant Surgery, Department of Surgery, Medical University of Graz, Auenbruggerplatz 29, 8036, Graz, Austria

**Keywords:** Liver disease, Transplantation, Long-term survival, Ability to work, Quality of life

## Abstract

**Background:**

Health-related quality of life (HrQoL) and workability are related parameters to measure success of therapy. Both have been insufficiently explored in patients after liver transplantation (LT). Particularly little is known about patients’ attitude to return to work, employment status before LT, and how frequently there is any employment at any time after LT.

**Methods:**

This is a single-center retrospective cohort study including 150 adult outpatients after LT. Liver transplantations had been performed between 1993 and 2018. The study was carried out from February to July 2018. The exclusion criteria were combined transplantations, positive screening for current alcohol abuse, and anxiety or depression. To evaluate HrQoL and fitness to work, the patients were tested using the Short Form 36, the Chronic Liver Disease Questionnaire, and the Work Ability Index.

**Key results:**

The return rate of sufficiently filled-in questionnaires was 46.8% (66 patients). The mean age of patients was 59.9 years (SD=10.8), ranging from 25 to 78 years old. HrQoL was partly comparable to the normal population. Workability sum scores with a mean value of 31.61 (SD 9.79) suggested moderate workability at present. While only 28.8% of respondents were ever employed after LT, 45.5% currently wished to work or would have wished to work.

**Conclusions:**

HRQL seems to be partly similar to population data, and subjective workability seems to be moderate in patients after LT. Despite a positive attitude to return to work in almost half of respondents, a lower rate of actual return to work was found in this study.

## Introduction

### Background

Liver transplantation (LT) is the treatment of choice for patients with terminal liver disease [1]. It has achieved excellent results, with a 1-year patient survival rate of up to 97% [2]. Therefore, further research in transplantation medicine has increasingly focused on long-term survival and health-related quality of life (HrQoL).

Two systematic reviews have confirmed improvement of HrQoL after LT compared to preoperative status [3, 4]. This improvement seems to persist in the long term as overall quality of life scores remain higher up to 20 years afterward, and the psychosocial component even comes close to approximating the data reported for the healthy population [4]. Nevertheless, despite marked improvement compared to the preoperative status, physical functioning continues to be inferior to the general population [3–5]. These general findings of gain in HrQoL after LT may not extend to all patient groups, e.g., to patients with hepatocellular carcinoma (HCC). This is possibly due to better pre-LT quality of life reported by these patients compared to those without HCC [6]. While most reports suggest similar results in HrQoL after heart, lung, kidney, and liver transplantation [7–9], there is one study [10] indicating the lowest quality of life benefits after LT [10]. An active coping style including participation in social life and work has increasingly been proposed for further improvement of HrQoL and medical outcome after LT [10–12]. Return to work (RTW) after LT has been found to be associated with better HrQoL [10, 13]. Employment after kidney transplantation has been reported to be linked with both overall recipient and graft survival in addition to better HrQoL [14]. However, there may be a discrepancy between working ability and the actual rate of employment after solid organ transplantation [15, 16]. In a study on working ability, only 4% of patients after kidney transplantation, 6% after LT, and 10% of patients after heart transplantation were described as unfit for any job based on an evaluation by the International Classification of Functioning, Disabilities, and Health (ICF) questionnaire [15]. In contrast, reported employment rates vary widely, with a range between 23 and 61% after LT [3, 10, 15], suggesting that substantially more patients could participate in paid employment after LT and return to some level of work than they currently seem to do. This may partly be due to health-related problems after LT, in particular fatigue and weakness [17]. The concept of subjective workability is determined by the demands of the job, on the one hand, and the individual’s health status, skills, and education, on the other, and can be measured by the Work Ability Index (WAI) [18, 19]. Further exploration of subjective workability after LT could contribute to better understanding the suggested discrepancy between subjective fitness to work and actual reintegration into working life [15].

### Objectives

Subjective workability and return to work have been scarcely studied in patients after LT, although both might impact on HrQoL. To help liver transplant recipients in this respect, the aim was to further explore the interrelations between HrQoL, workability, and employment after LT. Therefore, the primary objective of this study was to quantify generic and specific HrQoL after LT and to explore workability and employment status before and after LT. As a second objective, the study investigated the correlation of age, gender, highest level of education, employment status, attitude to RTW, indication for LT, and time since transplantation with HrQoL and workability.

## Methods

### Study design and setting

This is a single-center retrospective cohort study, conducted at the General, Visceral and Transplant Surgery, Dept. of Surgery, Medical University of Graz. The ethics committee of the Medical University of Graz has approved the study (ethics committee number 29-622 ex 16/17).

### Patients

**Fig. 1:**
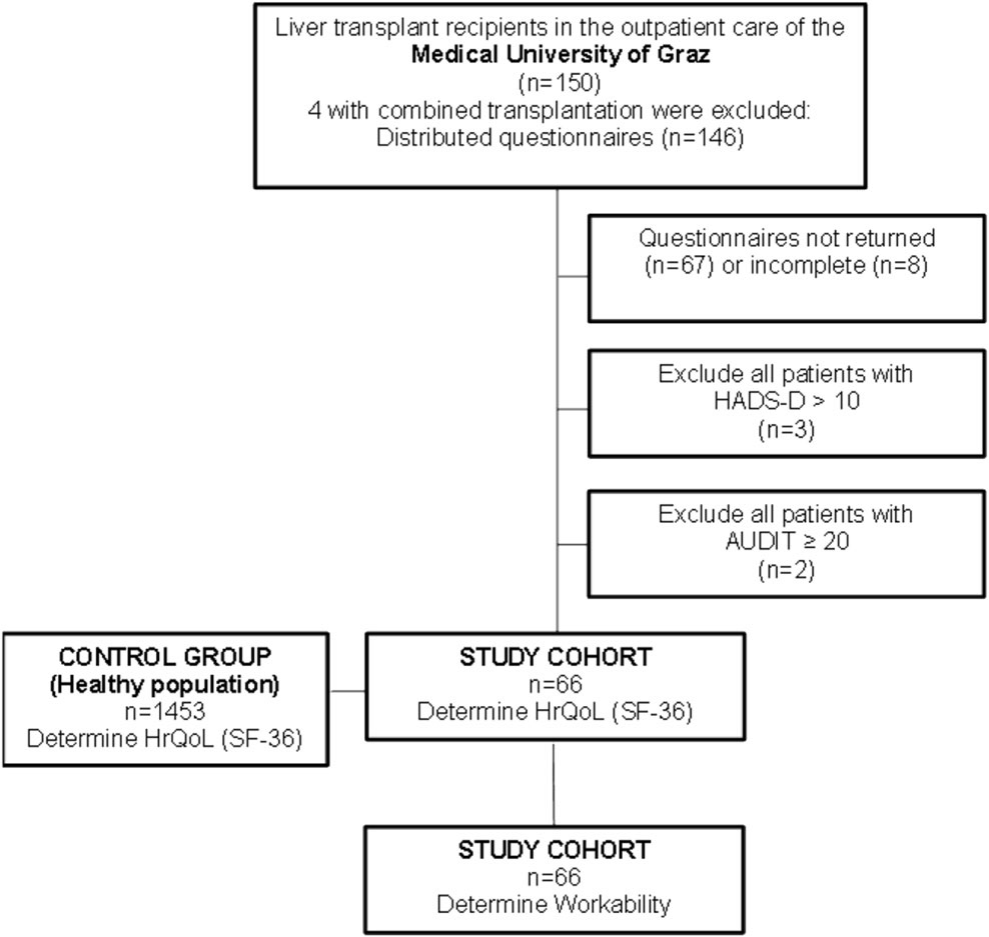
Return rate of sufficiently filled-in questionnaires

During their regular visit to the transplant outpatient clinic, adult outpatients after LT were consecutively invited to participate in the study (*n*=150). Liver transplantation in these patients had been performed between 1993 and 2018. The study was carried out from February to July 2018. The following exclusion criteria were applied in screening these patients for participation in this questionnaire study: combined transplantations (*n*=4), patients with current positive screening for alcohol abuse (AUDIT) (*n*=2), and current anxiety or depression (HADS-D) (*n*=3). Current alcohol abuse and current anxiety or depression were chosen as exclusion criteria to test for workability only in those patients after LT who were not additionally challenged by these prevalent mental health problems. The return rate of sufficiently filled-in questionnaires was 46.8% (66 patients) (Fig. 1).

### Variables

The following variables were evaluated: age, gender, highest level of educational background, living situation, indication for LT, and time after LT. With regard to occupational background, status of employment before LT, any employment after LT, and occupational status at present were explored. In addition, patients’ attitude towards RTW was investigated.

### Age

Patients were assigned to a total of three age groups: group 1, 50 years old and younger; group 2, between 51 and 60 years old; and group 3, 61 years old and older. The age division corresponds to the division of the Short Form 36 (SF-36).

### Questionnaires

#### Hospital Anxiety and Depression Scale (HADS-D)

The HADS-D is a standardized questionnaire that is commonly used to screen for depression and anxiety in patients with physical illnesses and complaints [20]. It consists of fourteen items that address anxious or depressed moods during the past week. These are rated on a 4-point scale, thus resulting in two sub-scores for anxiety and depression. Questionnaires from patients who achieved a value higher than 10 points on the HADS-D were considered a positive screening for anxiety or depression and therefore excluded from further data evaluation [20].

#### Alcohol Use Disorders Identification Test (AUDIT)

The AUDIT, developed by the WHO, is a screening tool for the identification of people with pathological alcohol consumption [21, 22]. The test consists of ten items, three concerning alcohol consumption or alcohol addiction, and four items that relate to alcohol abuse. A total score is calculated from all questions with possible values between 0 and 40. A score of ≥ 8 reveals problematic drinking behavior with reference to alcohol addiction. An AUDIT score of 20 or more warrants further diagnostic evaluation for alcohol dependence; therefore, we applied this threshold as the exclusion criterion for further data evaluation in this study [21, 22].

#### Short Form 36 (SF-36)

The 36-Item Short Form Health Survey (SF-36) is a disease non-specific multidimensional questionnaire assessing various aspects of health-related quality of life [23]. It examines both psychological and physical aspects of HrQoL. The SF-36 consists of 36 questions, which can be assigned to eight dimensions of subjective health. These are as follows: PF, physical functioning; RP, role physical; BP, bodily pain; GH, general health; VT, vitality; SF, social functioning; RE, role emotional; and MH, mental health. These eight dimensions can be mapped in a physical component summary (PCS) and a mental component summary (MCS), whereby PF, RP, and BP are typically assigned to the PCS, and MH, RE, and SF more to the MCS. Raw scores are transformed into scales ranging from 0 to 100, with 0 corresponding to the worst and 100 corresponding to the best possible HrQoL [24].

#### Chronic Liver Disease Questionnaire (CLDQ-D)

The CLDQ-D is a disease-specific questionnaire to evaluate the health-related quality of life in people with chronic liver disease [25]. The questionnaire consists of 29 questions which assess the following six disease-related categories: fatigue (5 questions), activity (3 questions), emotional function (8 questions), abdominal symptoms (3 questions), systemic symptoms (5 questions), and worry (5 questions). The test is evaluated by determining a total score for HrQoL and scores for the six subscales, with values ranging between 1 and 7. The total value for HrQoL is calculated as a sum score of all items and ranges between 29 and 203 points, with lower values reflecting a poorer quality of life. The CLDQ-D measurement correlates with the severity of the liver disease [25].

#### Work Ability Index (WAI)

From an occupational health perspective, workability as measured by the WAI is determined by the demands of the job, on the one hand, and the individual’s health status, skills, and education, on the other [18]. This test evaluates the subjectively perceived ability to work, with the frequent aim to maintain, restore, and promote work ability [19]. The WAI consists of ten questions which cover physical and psychological work requirements, the state of health, and the personal performance reserves. The items can be allocated to seven dimensions, and overall scores can range between 7 and 49. Workability is reflected by the sum score and can be interpreted as poor [7–27], moderate (28–36), good (37–43), or very good (44–49) [19].

### Healthy reference population for comparison of SF-36 values

Comparative values of a healthy population are available in the SF-36 manual. It contains data from 1453 healthy persons (661 males, 792 females) [26]. The age distribution is categorized into three groups: 50 years old or younger (54.6%), 51 to 60 years old (18.7%), and 61 years old or older (26.5%). The average age of the comparison population is 42 years old. To compare the results of this healthy reference population with those after LT, median comparisons with mean-imputed values were carried out.

### Statistical methods

Statistical analysis was done using Microsoft Excel 2016 and IBM SPSS Statistics Versions 22 and 26. Due to the unknown distribution of the healthy control group’s data in the SF-36, comparisons of medians between patients after LT and healthy controls were calculated by Wilcoxon signed-rank tests. Shapiro-Wilk tests for normality showed that most continuous variables were not normally distributed within our sample. In these cases, differences between patient subgroups in quality of life and workability were detected by Mann-Whitney *U* tests for gender and by Kruskal-Wallis tests for all variables with three or four factor levels (age, indications for LT, time passed after LT, and employment groups). Statistical significance was assumed at a *p*-value of <0.05.

## Results

### Demographics

Sixty-six patients after LT (52 male, 14 female) were included in the analysis. The mean age of patients at present was 59.9 years old (SD=10.8), ranging from 25 to 78 years old. Details on demographic and clinical characteristics are displayed in Table 1.

**Table 1:** Demographics and group characteristics

		*n* (%)*
Gender	Male	52 (78.8)
	Female	14 (21.2)
Age	≤ 50 years	11 (16.7)
	51–60 years	18 (27.3)
	≥ 61 years	37 (56.1)
	(mean, SD)	59.9 (10.8)
Education	Primary education	10 (15.2)
	Secondary education	37 (56.1)
	Post-secondary/university	12 (18.2)
	Unclear	7 (10.6)
Employment status	Full time	16 (24.2)
	Part time	1 (1.5)
	Housewife/student	3 (4.5)
	Retirement pension	14 (21.2)
	Early retirement	20 (30.3)
	Other	5 (7.6)
	Unclear	7 (10.6)
Living situation	Alone	15 (22.7)
	With partner/family	44 (66.7)
	Unclear	7 (10.6)
Indication for LT	ALD	25 (37.9)
	HCC	5 (7.6)
	HCV	7 (10.6)
	Other	24 (36.4)
	Unclear	5 (7.6)
Time passed after LT	< 5 years	29 (43.9)
	5–15 years	28 (42.4)
	> 15 years	4 (6.1)
	Unclear	5 (7.6)

*Numbers may not sum to 100% because of rounding

*LT* liver transplantation, *SD* standard deviation, *ALD* alcoholic liver disease, *HCC* hepatocellular carcinoma, *HCV* hepatitis C virus-associated

#### Clinical characteristics

Patients were divided into four categories of former indications for LT: ALD (alcoholic liver disease, *n*=25), HCC (hepatocellular carcinoma, *n*=5), HCV (hepatitis C virus-associated, *n*=7), and other indications including primary and secondary sclerosing cholangitis, primary biliary cholangitis, hepatitis B virus-associated liver cirrhosis, hemochromatosis, autoimmune-hepatitis, alpha-1-antitrypsin deficiency, and acute liver failure (*n*=24) (Table 1).

#### Employment status

In addition to employment status at present (Table 1), the employment status before LT, and if there was any employment after LT at any time, was also explored (Table 2).

**Table 2:** Employment status before and after LT and attitude towards employment

		*n* (%)*
Employment status before LT	Full time	30 (45.5)
	Housewife/student	3 (4.5)
	Retirement pension	10 (15.2)
	Early retirement	11 (16.7)
	Other (sick leave, unemployed, school)	4 (6.1)
	Unclear	8 (12.1)
Ever being employed after LT	Yes	19 (28.8)
	No	40 (60.6)
	Unclear	7 (10.6)
Employment status if ever employed after LT	Full time	15 (22.7)
	Part time	3 (4.5)
	Other	1 (1.5)
If never employed after LT, reason for not being employed	Retirement pension before LT	12(18.2)
	Retirement pension shortly after LT	3 (4.5)
	Early retirement due to liver disease	14 (21.2)
	Early retirement due to other disease	6 (9.1)
	Health reasons without retirement	1 (1.5)
	Own wish	1 (1.5)
	Unemployment	2 (3.0)
	Sick leave	1 (1.5)
Wish to work or would have worked after LT	Yes	30 (45.5)
	No	28 (42.4)
	Unsecure	1 (1.5)
	Unclear	7 (10.6)

*Numbers may not sum to 100% because of rounding

*LT* liver transplantation

### Generic HrQoL after LT

The median of “physical health” (PCS) at present was 50.17 (IQR 9.31) and 50.19 (IQR 13.13) for “mental health” (MCS) (Table 3). Only values for PCS, but not for MCS, are significantly below the healthy population (Table 3). Independent of the patients’ age, current HrQoL in liver recipients was worst for the dimensions “role physical” and “social functioning” (Table 3). In contrast, the sub-scores for vitality and mental health did not reveal any differences between patients after LT and healthy controls (Table 3).

**Table 3:** HrQoL after LT in comparison to healthy controls

SF-36 scales	Age groups	LT patients		Healthy controls	Statistics (Wilcoxon signed-rank test)	
		Mean (SD)	Median (IQR)	Median (IQR)	*Z*	*p*
Physical component summary	Total	49.94 (8.50)	50.2 (9.3)	55.4 (7.8)	378.000	.000**
	≤ 50 years	48.29 (9.34)	49.5 (7.9)	56.4 (5.7)	5.000	.013*
	51–60 years	49.32 (9.25)	51.8 (17.3)	53.8 (9.9)	51.000	.133
	≥ 61 years	50.73 (8.01)	49.5 (8.3)	49.9 (13.3)	421.000	.293
Mental component summary	Total	50.85 (8.30)	50.2 (13.1)	53.3 (7.9)	830.000	.078
	≤ 50 years	52.54 (7.71)	51.5 (11.7)	52.8(7.6)	33.000	1.000
	51–60 years	50.94 (9.00)	52.0 (18.3)	53.7 (8.8)	55.000	.184
	≥ 61 years	50.31 (8.27)	50.2 (11.0)	54.9 (7)	159.000	.004**
Physical functioning	Total	74.43 (26.94)	85 (40)	100 (10)	.000	.000**
	≤ 50 years	76.36 (26.56)	95 (50)	100 (5)	.000	.011*
	51–60 years	76.67 (25.61)	90 (40)	95 (15)	6.000	.005**
	≥ 61 years	72.50 (28.45)	82.5 (47.5)	85 (30)	131.500	.061
Role physical	Total	64.05 (42.91)	99.5 (93.8)	100 (0)	.000	.000**
	≤ 50 years	77.27 (30.53)	75 (25)	100 (0)	.000	.024*
	51–60 years	56.94 (43.56)	50 (100)	100 (0)	.000	.004**
	≥ 61 years	63.54 (45.89)	100 (100)	100 (50)	.000	.000**
Bodily pain	Total	78.51 (27.79)	99.5 (43.1)	100 (28)	.000	.000**
	≤ 50 years	82.05 (22.63)	100 (32.5)	100 (26)	.000	.041*
	51–60 years	75.97 (29.90)	93.8 (55)	100 (48)	.000	.008**
	≥ 61 years	78.69 (28.89)	99 (38.8)	84 (38.8)	345.000	.921
General health	Total	69.79 (19.87)	70 (35)	77 (25)	661.000	.027*
	≤ 50 years	65.91 (16.71)	65 (20)	77 (20)	11.000	.049*
	51–60 years	72.61 (20.66)	73.5 (41.3)	67 (25)	116.000	.183
	≥ 61 years	69.55 (20.70)	70 (35)	67 (25)	327.000	.406
Vitality	Total	66.95 (20.92)	70 (36.3)	70 (25)	804.000	.308
	≤ 50 years	65.91 (17.58)	70 (30)	70 (15)	17.000	.513
	51–60 years	65.00 (21.90)	67.5 (42.5)	70 (25)	43.000	.334
	≥ 61 years	68.21 (21.78)	73.3 (35)	65 (30)	349.500	.372
Social functioning	Total	81.35 (20.68)	87.5 (37.5)	100 (12.5)	.000	.000**
	≤ 50 years	78.75 (20.45)	87.5 (40.6)	100 (12.5)	.000	.017*
	51–60 years	81.94 (17.79)	81.25 (28.13)	100 (12.5)	.000	.003**
	≥ 61 years	81.79 (22.55)	87.5 (25)	100 (12.5)	.000	.000**
Role emotional	Total	79.14 (38.24)	100 (25.3)	100 (0)	.000	.000**
	≤ 50 years	81.82 (40.45)	100 (0)	100 (0)	.000	.157
	51–60 years	75.93 (39.28)	100 (41.7)	100 (0)	.000	.026*
	≥ 61 years	79.94 (38.06)	100 (1)	100 (0)	.000	.004**
Mental health	Total	80.11 (15.07)	80 (26.3)	76 (20)	1249.000	.029*
	≤ 50 years	77.45 (15.52)	80 (16)	76 (16)	33.500	.538
	51–60 years	78.33 (15.50)	80 (22)	76 (20)	103.500	.431
	≥ 61 years	81.86 (14.93)	84 (28)	80 (20)	310.000	.388

*p < .05, **p < .01

*SD* standard deviation, *IQR* interquartile range, *LT* liver transplantation, *SF-36* 36-Item Short Form Health Survey

#### Differences according to age groups and comparison to healthy controls

In a comparison of age subgroups between patients after LT and the healthy control group of the same age, younger patients generally reported having worse physical health and older patients reported having worse mental health than healthy controls (Table 3). Interestingly, patients in the middle age group (51–60 years old) reported neither a significantly worse physical state nor mental state of health compared to their peers in the healthy reference population.

#### Gender-related differences

When comparing male and female patients in terms of the SF-36, female patients after LT reported lower quality of life in the “role emotional” (*U*=210.5, *p*=0.011) and “physical health (PCS)” (*U*=231.0, *p*=0.037) scales.

#### Differences in HrQoL according to employment status among patients after LT

To investigate differences in HrQoL according to employment status, patients after LT were split into three groups: participants who were currently working full time, part time, at home, or studying were considered as “employed” (*n*=20, 30.3%); retired patients formed the second group (*n*=14, 21.2%); and the third group consisted of patients who were unable to work due to early retirement, sick leave, or unemployment (*n*=25, 37.9%). Group comparisons between these three employment groups showed that two dimensions of HrQoL, in particular, were associated with employment status (Table 4): physical functioning and, in a statistical tendency, social functioning. Physical functioning was higher in employed participants as compared to patients in early retirement, sick leave, or unemployment (post hoc analysis according to Dunn-Bonferroni: *Z*=13.10, *p*=0.027). Concerning social functioning, there was a statistical tendency that patients in early retirement, sick leave, or unemployment had lower HrQoL than employed patients or retired patients (Table 4).

**Table 4:** Differences in HrQoL according to employment status

SF-36 scales	Employment groups	Mean (SD)	Mean rank	Statistics (Kruskal-Wallis test)	
				*H*	*P*
Physical component summary	Employment	50.45 (9.95)	27.22		
	Retirement pension	51.69 (9.25)	29.21		
	Early ret./sick/unemp.	47.62 (9.66)	22.47	1.85	.397
Mental component summary	Employment	53.88 (8.21)	29.67		
	Retirement pension	49.84 (7.00)	22.93		
	Early ret./sick/unemp.	49.93 (10.55)	24.79	1.82	.403
Physical functioning	Employment	87.63 (18.06)	36.42		
	Retirement pension	72.14 (25.02)	26.25		
	Early ret./sick/unemp.	66.30 (29.20)	23.33	7.21	.027*
Role physical	Employment	72.37 (38.09)	31.39		
	Retirement pension	60.71 (44.63)	28.71		
	Early ret./sick/unemp.	59.33 (45.89)	27.27	.76	.683
Bodily pain	Employment	84.63 (25.61)	34.45		
	Retirement pension	75.54 (28.52)	27.68		
	Early ret./sick/unemp.	74.76 (31.49)	27.74	2.38	.304
General health	Employment	74.05 (19.88)	32.61		
	Retirement pension	70.36 (19.46)	28.25		
	Early ret./sick/unemp.	67.50 (19.73)	26.58	1.44	.486
Vitality	Employment	71.87 (18.79)	33.38		
	Retirement pension	68.21 (19.18)	30.79		
	Early ret./sick/unemp.	62.49 (23.76)	26.86	1.65	.439
Social functioning	Employment	88.16 (15.29)	32.63		
	Retirement pension	88.39 (11.46)	31.86		
	Early ret./sick/unemp.	72.83 (26.29)	23.04	4.82	.090(*)
Role emotional	Employment	84.21 (37.46)	32.87		
	Retirement pension	73.81 (43.71)	29.21		
	Early ret./sick/unemp.	72.14 (40.07)	25.81	2.85	.241
Mental health	Employment	81.85 (17.76)	32.45		
	Retirement pension	82.29 (14.61)	31.43		
	Early ret./sick/unemp.	77.75 (14.11)	25.92	1.89	.388

*p < .05, (*).1>*p*>.05

*SD* standard deviation, *SF-36* 36-Item Short Form Health Survey

### Liver disease-specific HrQoL

For the total value of the CLDQ-D measurement, the cohort showed a median of 170.0 (IQR 29.3), which demonstrates a limitation of HrQoL of 18.96% (Table 4).

The results of the individual dimensions of the CLDQ-D showed that female patients after LT have lower HrQoL concerning systemic symptoms than male patients (*U*=206.5, *p*=0.043). With regard to all other dimensions of their disease-specific HrQoL, no differences between genders could be found (*p*>0.05, data not shown). Patients in the three different age groups did not differ in their disease-specific quality of life as measured by the CLDQ-D (*p*>0.05, data not shown). Age does not appear to have any influence on the overall index or individual categories (Table 5).

**Table 5:** Disease-specific HrQoL after LT

CLDQ-D	Mean (SD)	Median (IQR)
Sum score	168.91 (29.47)	170.0 (29.3)
Fatigue	5.52 (1.17)	5.8 (1.77)
Activity	5.80 (1.22)	6.0 (1.58)
Emotional function	5.96 (1.14)	6.25 (1.38)
Abdominal symptoms	5.86 (1.23)	6.0 (1.67)
Systemic symptoms	5.87 (1.06)	6.0 (1.2)
Worry	6.18 (1.24)	6.6 (1.2)

*SD* standard deviation, *IQR* interquartile range, *CLDQ-D* Chronic Liver Disease Questionnaire, German version

### Workability

The total workability score, with a mean value of 31.61 (SD 9.79), is within the range of subjectively perceived moderate ability to work (28–36). The results of all seven dimensions of the WAI are shown in Table 6. No gender differences were found with regard to the current ability to work, neither in the overall index nor in the individual dimensions (*p*>0.05, data not shown).

**Table 6:** Workability of patients after LT in total and according to employment status

WAI dimensions	Employment groups	Mean (SD)	Mean rank	Statistics (Kruskal-Wallis test)	
				*H*	*P*
Dimension 1: Current work ability compared with lifetime best	Total	6.07 (2.75)			
	Employment	6.94 (2.88)	30.85		
	Retirement pension	6.27 (2.05)	26.68		
	Early ret./sick/unemp.	5.30 (2.98)	22.09	3.49	.175
Dimension 2: Work ability in relation to the demands of the job	Total	6.98 (2.24)			
	Employment	7.54 (2.37)	25.38		
	Retirement pension	7.13 (1.96)	22.25		
	Early ret./sick/unemp.	6.29 (2.22)	18.81	2.41	.300
Dimension 3: Number of current diseases diagnosed by a physician	Total	3.10 (1.97)			
	Employment	3.26 (1.94)	29.16		
	Retirement pension	3.85 (2.15)	33.23		
	Early ret./sick/unemp.	2.45 (1.47)	22.68	4.29	.117
Dimension 4: Estimated work impairment due to diseases	Total	4.52 (1.79)			
	Employment	4.72 (1.72)	29.14		
	Retirement pension	4.46 (1.61)	25.73		
	Early ret./sick/unemp.	4.27 (2.00)	26.00	.58	.748
Dimension 5: Sick leave during the past 12 months	Total	4.00 (1.49)			
	Employment	3.94 (1.47)	23.92		
	Retirement pension	4.36 (1.29)	28.82		
	Early ret./sick/unemp.	3.75 (1.71)	23.88	1.26	.531
Dimension 6: Personal prognosis of work ability 2 years from now	Total	5.42 (2.30)			
	Employment	5.83 (2.09)	28.25		
	Retirement pension	5.91 (2.03)	28.55		
	Early ret./sick/unemp.	4.57 (2.62)	21.55	3.56	.169
Dimension 7: Mental resources	Total	3.08 (0.99)			
	Employment	3.21 (1.08)	30.53		
	Retirement pension	3.00 (0.78)	25.36		
	Early ret./sick/unemp.	3.05 (1.05)	27.50	.99	.608
Total score workability	Total	31.61 (9.79)			
	Employment	34.04 (10.65)	34.38		
	Retirement pension	31.06 (8.50)	28.50		
	Early ret./sick/unemp.	29.95 (10.12)	27.34	2.01	.366

*SD* standard deviation, *WAI* Work Ability Index, *early ret./sick/unemp.* early retirement, sick leave, unemployment

With regard to the overall index of the questionnaire, the age of the patients played no role in their ability to work (Kruskal-Wallis *H*=2.214, *p*=0.330). When considering the individual dimensions of workability, significant differences (Kruskal-Wallis *H*=8–734, *p*=0.013) could be found for dimension 5 (sick leave during the past 12 months): the older the patient, the fewer days absent. The other individual dimensions showed no significant age-specific differences (Table 6).

### Attitude to return to work, workability, and HrQoL

As a next step, we wanted to compare subjective workability and quality of life at present among three groups of patients: (i) patients who wanted to work after LT and succeeded in working (*n*=19, 28.8%), (ii) those who wanted to work but could not for several reasons (*n*=11, 16.7%), and finally (iii) the group who did not want to work after LT and also did not work (*n*=28, 42.4%). Workability among the first two groups was similar (*M*=34.9, SD=10.8 and *M*=33.9, SD=8.0), whereas the mean score of group 3 was several points lower (*M*=28.6, SD=11.2). Although these differences did not achieve statistical significance (Kruskal-Wallis *H*=3.26, *p*=0.196), a score of 28.6 according to the WAI classification would correspond to the bottom of the category of moderate workability (a score of 27 is already classified as “critical” workability), whereas scores of 35 and 36 belong to the upper end of moderate workability (a score of 37 is already classified as “good” workability).

Concerning quality of life as measured by the sum scores of SF-36 and CLDQ-D, these three groups did not differ from each other (*p*>0.05, data not shown).

## Discussion

This study was designed to investigate HrQoL, workability, employment status, and their interrelation in patients after LT, as there has been scant research to date on workability and RTW after LT, in particular.

In accordance with findings in the literature, study participants reported moderately reduced HrQoL, primarily in terms of the physical component as compared to healthy controls [3–5]. Independent of the patients’ age, the most substantial deficits, based on SF-36, were found in the dimensions of “role physical” and “social functioning.” A comparison with the healthy reference population according to gender revealed that deficits in “role physical” were primarily reported by women. Both sexes similarly reported deficits in social functioning, which imply reduced social contact. These deficits may deserve additional attention as they tend to be accompanied by reduced social support and reduced social wellbeing and, in addition, may negatively influence patient-provider interaction and adherence to treatment [27–30]. Interestingly, with regard to age groups, younger participants reported having worse physical health, and older patients reported having worse mental health than healthy controls in a comparison of SF-36 data.

Neither the length of time passed after LT nor indications for LT had an effect on the quality of life in this study cohort. With regard to ALD and HCV as indications for LT, this may be somewhat surprising, as worse HrQoL after LT has been repeatedly reported for patients with these indications [3, 4, 31]. In the case of ALD, this may be due to personalized support of these patients in outpatient care, consisting of regular checks and increased measures, if necessary. In the case of HCV, similar HrQoL after LT compared to other indications may reflect new treatment options of hepatitis C in recent years, leading to improved medical outcome, including better HrQoL.

Besides HrQoL, maintenance of employment or RTW has been gaining increasing attention in organ transplantation in general, due to the widely established long-term survival [13–17]. Employment in working-age patients is regarded as an important part of returning to “full-life” participation [12]. As has been shown for kidney transplant recipients, additional advantages of RTW for HrQoL [30] and even for patient and graft survival [14] can be expected. In addition, for different organ transplantations, a discrepancy between workability and actual RTW has been assumed [15, 16]. The results of this study also point in this direction for liver transplant recipients.

However, when social issues such as RTW after surgery are studied, the impact of the national social security system on the patient’s attempt to retain a job or RTW and the role of other stakeholders, especially the employer’s role, need to be considered. In Austrian social legislation, based on the subsequent need for lifelong immunosuppression, an “earning capacity reduction” status is granted even if there is an excellent medical outcome after LT. This disability status is targeted at workers with chronic diseases and is associated with some extent of legal protection, resulting in social and vocational benefits. Although disability status provides relevant support regarding (re)employment, patients may lose their jobs before this status is granted, e.g., during long periods of sick leave, and it may become particularly difficult to get hired again.

On average, study participants reported moderate workability according to WAI measurement, which seems sufficient to consider RTW accordingly. It should be mentioned that WAI is typically applied in occupational medicine to test for difficulties in working life, with the aim of providing preventive interventions to ensure that working life can be maintained [32]. Furthermore, almost half of respondents (45.5%) expressed that they wished to work or would have wished to work. Interestingly, those who expressed a positive attitude to RTW also reported better values regarding workability, with a mean value close to “good workability,” though not quite statistically significant, as compared to those who did not wish to RTW. In contrast, less than a third of participants (28.8%) were ever employed again after LT. In this context, it should be noted that those who were involved in some sort of work (working full time, part time, at home, or studying) reported better physical functioning (*H*=7.21, *p*=0.027) and, in a statistical tendency, better social functioning (*H*=4.82, *p*=0.090).

These findings may suggest a discrepancy between the reported interest to RTW along with a sufficient self-perceived workability, on the one hand, and the possibility to RTW, on the other. This may deserve further attention as RTW can be assumed to be beneficial for long-term outcome, particularly with regard to HrQoL. Both individual and system-related reasons for such a discrepancy should be explored. It has been recently suggested that fatigue and weakness may hinder patient social reintegration after liver transplantation [17]. Although liver-related symptom load may be a burden for patients after LT, in our study, liver-related deficits in HrQoL as measured by the CLDQ-D were only reported to be about 19% less than optimal and did not prevent nearly half of respondents from expressing a positive attitude to RTW.

## Conclusion

In accordance with previous research in patients after LT, the findings of this study suggest a similar mental health component as compared to population data and a partly reduced physical health component of HRQL in this patient group. On average, subjective workability seems to be moderate in patients after LT. Despite a positive attitude to return to work in almost half of respondents, a lower rate of actual return to work was found in this study. To better understand this finding, the impact of individual and systemic factors which can influence attitudes to returning to work and actual RTW need to be considered. This may be crucial to better support patients after LT with regard to potentially unmet needs on this matter of interest in RTW and actual (re)employment. Therefore, a prospective multicenter study should be conducted to further analyze the interrelations between HrQoL, workability, attitude to RTW, and employment status prior to and after LT, which also allows social, cultural, and economic factors to be taken into account.
